# Simulated climate change conditions unveil the toxic potential of the fungicide pyrimethanil on the midge *Chironomus riparius*: a multigeneration experiment

**DOI:** 10.1002/ece3.71

**Published:** 2012-01

**Authors:** Ruth Müller, Anne Seeland, Lucas S Jagodzinski, Joao B Diogo, Carsten Nowak, Jörg Oehlmann

**Affiliations:** 1 LOEWE Biodiversity and Climate Research CentreSenckenberganlage 25, D-60325 Frankfurt am Main, Germany; 2 Department of Aquatic Ecotoxicology, Goethe UniversitySiesmayerstr. 70, D-60323 Frankfurt am Main, Germany; 3 School of Biological, Earth and Environmental Science, National University IrelandDistillery Fields, North Mall, Cork, Ireland; 4 Senckenberg Research Institute and Natural History MuseumClamecystraße 12, Gelnhausen, D-63571 Gelnhausen, Germany

**Keywords:** Dynamic temperature, genetic diversity, low-dose effects, multiple stressors, near-natural, NOAEC

## Abstract

Although it has been suggested that temperature increase may alter the toxic potential of environmental pollutants, few studies have investigated the potential risk of chemical stressors for wildlife under Global Climate Change (GCC) impact. We applied a bifactorial multigeneration study in order to test if GCC conditions alter the effects of low pesticide concentrations on life history and genetic diversity of the aquatic model organism *Chironomus riparius*. Experimental populations of the species were chronically exposed to a low concentration of the fungicide pyrimethanil (half of the no-observed-adverse-effect concentration: NOAEC/2) under two dynamic present-day temperature simulations (11.0–22.7°C; 14.0–25.2°C) and one future scenario (16.5–28.1°C). During the 140-day multigeneration study, survival, emergence, reproduction, population growth, and genetic diversity of *C. riparius* were analyzed. Our results reveal that high temperature and pyrimethanil act synergistically on the midge *C. riparius*. In simulated present-day scenarios, a NOAEC/2 of pyrimethanil as derived from a life-cycle toxicity test provoked only slight-to-moderate beneficial or adverse effects on *C. riparius*. In contrast, exposure to a NOAEC/2 concentration of pyrimethanil at a thermal situation likely for a summer under GCC conditions uncovered adverse effects on mortality and population growth rate. In addition, genetic diversity was considerably reduced by pyrimethanil in the future scenario, but only slightly under current climatic conditions. Our multigeneration study under near-natural (climatic) conditions indicates that not only the impact of climate change, but also low concentrations of pesticides may pose a reasonable risk for aquatic insects in future.

## Introduction

An increasing number of studies document currently observed effects of Global Climate Change (GCC) on aquatic biodiversity and numerous projections predict dramatic species loss and disrupted functioning, persistence, and resilience of many ecosystems due to fast-changing climatic conditions (Beznosov and Suzdaleva 2004; Mooij et al. 2005; Thackeray et al. 2010). In contrast, combined effects of GCC and additional stressors, such as environmental pollutants, are less understood. Ecological risk assessment (ERA) faces new challenges under altered environmental conditions such as GCC (Segner 2007; Wenning et al. 2010). The ecotoxicity of xenobiotics often alters at suboptimal conditions such as high temperature, but the direction and extent of the alterations are difficult to predict (Cairns et al. 1975; Mayer and Ellersieck 1986; Heugens et al. 2001; Oetken et al. 2009). Rarely undertaken multigeneration studies provide, moreover, evidence that effects of either toxicants or temperature alter during consecutive generations (Postma and Davids 1995; Bossuyt and Janssen 2004; Vogt et al. 2007a; Nowak et al. 2009; Salice et al. 2009). Nevertheless, the available unifactorial toxicity tests performed at constant temperatures appear to be too simplistic, as wild populations are successively exposed to suboptimal, optimal, and superoptimal temperatures during consecutive generations (Segner 2007). Near-natural temperature regimes may modify the response of aquatic species to toxicant stress in the long term, especially at high summer temperatures under GCC conditions.

The traditional ERA faces additional uncertainties regarding low-dose risk assessment (Postma and Davids 1995; Foran 1998; Sielken and Stevenson 1998; Calow and Forbes 2003; Segner 2007; Calabrese 2008, 2010; wenning et al. 2010). Population dynamics can be affected at even a regulatory approved no-observed-adverse-effect concentration (NOAEC) of a chemical if concerning multiple generations (Postma and Davids 1995). Moreover, at the concentration spectrum below the NOAEC, cellular stress responses may increase and reproduction may decrease (Roux et al. 1993; Damelin et al. 2000). Thus, low doses of xenobiotics below the adverse effect concentrations may be more risky than so far considered by ERA (Sielken and Stevenson 1998; Calabrese 2008, 2010), particularly under GCC conditions.

Therefore, in this study we aimed to investigate the combined effects of chronic low-dose pesticide exposure and GCC in a multigeneration study. The ecotoxicological model organism *Chironomus riparius* (Meigen 1804) was chosen as a test organism. This aquatic insect (Diptera, Chironomidae) species is a standard test organism in aquatic ERA (OECD 2004) and former research papers hint to complex effects of both chemical and thermal stressors on multigeneration population dynamics (Postma and Davids 1995; Vogt et al. 2007a, 2007c; Nowak et al. 2009). We hypothesized (1) that a low-dose (below NOAEC) concentration of the pesticide will show significant effects on life history and genetic diversity of *C. riparius* populations in long term, and (2) that disregarding seasonal temperature variation under current and potential GCC conditions will lead to false-negative estimations of the environmental risk of low doses of pesticides to aquatic species.

To test these hypotheses, we incipiently assessed the concentration–response relationship of a model fungicide, pyrimethanil, on *C. riparius* via a toxicity test lasting for one generation including reproductive endpoints for the investigation of population-level effects (OECD 2004). The multigeneration effect of a low fungicidal concentration (NOAEC/2) as derived from the life-cycle toxicity test was subsequently investigated under a typical temperature situation for cold-temperate watercourses (slow flowing or nonstratified) in spring and summer of (1) a cold year in 1990–2005, (2) a warm year in 1990–2005, and (3) a warm year expected for 2070–2100. During the multigeneration study, parameters related to survival, emergence, reproduction, population growth, and genetic diversity were analyzed.

## Materials and Methods

### Life-cycle toxicity test

To obtain a concentration–response relationship for pyrimethanil (4,6-dimethyl-N-phenyl-2-pyrimidinamine), first-instar larvae (L1-larvae) of *C. riparius* (laboratory-maintained culture at Goethe University, Frankfurt am Main, Germany) were exposed for one generation to control/solvent control conditions and to six nominal pyrimethanil concentrations (2, 4, 8, 16, 24, and 32 mg×L^–1^ Pestanal®, analytical standard (Fluka), Sigma-Aldrich, Taufkirchen, Germany).

Pyrimethanil was adopted as model fungicide due to several experimentally advantageous characteristics, such as the low risk for human health, the moderate water solubility and degradation time, the negligible degradation via hydrolysis and photolysis, and the ease of sound quantification (EFSA 2006). Pyrimethanil is often appliquéd in vineyards (≤1 kg×ha^–1^, once a year), apple orchards, and protein pea cultures (≤600 g×ha^–1^, 2–3 times per year, EFSA 2006). The predicted environmental concentration of pyrimethanil in surface waters (PEC_sw_) accounts to ≤90 μg×L^–1^ for apple orchards and ≤27 μg×L^–1^ for vine cultures (EFSA 2006). In accordance, pyrimethanil was frequently detected in European surface waters at concentrations up to 22 μg×L^–1^ (Schlichtig et al. 2001; Verdisson et al. 2001; Kreuger et al. 2010). It has however to be noted, that high amounts of pyrimethanil are regularly released from sewage plants into the aquatic environment (up to 200 g×week^–1^ in 1999–2000, up to 400 g×week^–1^ in 2006, up to 80 g×week^–1^ in 2007), in particular during summer months (Schlichtig et al. 2001; Blarr 2008).

The test was carried out according to OECD guideline 219 (OECD 2004). Each treatment consisted of five replicates for biological analysis and one replicate for weekly physical/chemical measurements. Midges were exposed in an environmental chamber to 20 ± 1.5°C and 60% humidity for 28 days. Light was provided by daylight® tubes (18 W/840, Osram, München, Germany) and set to 1800 Lux and a 16:8 h light:dark cycle. Four days prior experimental start, 10 freshly laid egg ropes were separated from stock culture into 24-well plates (2 mL reconstituted water [conductivity 537 μS×cm^–2^, pH 8.4] per well).

Hatched larvae were pooled and L1-larvae were randomly inserted into 2-L quartz glass test vessels filled with aged sediment (5 days) and 1 L reconstituted water. The sediment consisted of washed and sterilized (24 h at 220°C) quartz sand (QuickMix®, quick-mix Gruppe, Osnabrück, Germany) with following granulometry: 0.1% > 500 μm, 34.3% > 250 μm, 50.0% > 150 μm, 10.6% > 125 μm, 5.0% > 63 μm, and 0.03% > 20 μm. In addition, sediment contained 0.4% handpicked leaves of *Fagus sylvatica* (particle size <500 μm; wetted with 111 mL reconstituted water per gram). Subsequent placement of the L1-larvae (60 per vessel, 0.1 larvae×cm^–2^), either 200-μl reconstituted water (control), 200-μl ethyl acetate (solvent control), or 200 μl of pyrimethanil solved in ethyl acetate (six levels) were added and gently dispersed. One day after application, vessels were slightly aerated and covered with gauze (mesh size 1.5 mm). Every other day, larvae were fed with finely grounded fish food (Tetra Min®, Tetra GmbH, Melle, Germany) with 0.25 mg×larvae^–1^×day^–1^ (day 1–5), 0.5 mg×larvae^–1^×day^–1^ (day 6–11) and 1 mg×larvae^–1^×day^–1^ (from day 12 onwards).

Number and sex of emerged adults and dead pupae were daily monitored, and all adults of one replicate were transferred with an exhauster to individual breeding cages (20 × 20 cm × 15 cm). A glass dish filled with approximately 50-mL reconstituted water was placed into each breeding cage to allow for oviposition. Water in oviposition containers was renewed every second day. Eggs were daily collected into 24-well plates filled with 2-mL reconstituted water per well. Temperature, oxygen saturation, and pH (CellOx 325, SenTix 11, WTW, Weilheim, Germany) were weekly measured in the extra test vessels and at the beginning and end of the test in the biological replicates. Conductivity (TetraCon 325, WTW) was examined at the beginning and at the end of experiment, other than nitrite, ammonium, and phosphate (Aquaquant, Merck, Darmstadt, Germany) measured at the end of the experiment (day 29).

### Multigeneration experiment

To estimate the response of *C. riparius* to combined thermal and low-dosed fungicide pollution under current conditions and a future climate change scenario, we performed a multigeneration experiment over a duration of 140 days (four to five generations). L1-larvae of *C. riparius* were exposed to 2-mg pyrimethanil L^–1^ at the beginning of each generation. The multigeneration study was conducted in environmentally controlled cabinets (MKKL 1200, Flohrs Instruments GmbH, Utrecht, Netherlands) to implement three dynamic temperature treatments. Dynamic temperature regimes represented a typical cold year in 1990–2005 (cold year–CY), a warm year in 1990–2005 (warm year–WY), and a temperature regime expected for a warm year in 2070–2100 (future warm year–WYF).

**Figure 1:**
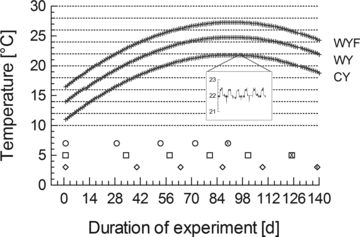
Thermal settings (lines), measured daily temperature variance (inset), and life-cycle duration of consecutive *C. riparius* generations (symbols) during the multigenerational study. CY = simulation of a cold growing season in 1990–2005; squares = WY exposure; WY = simulation of a warm season in 1990–2005; circles = WYF exposure; WYF = simulation of a projected warm season in 2050–2080; open symbols = start of generations; crossed symbols = end of final generations; diamonds = CY exposure.

Water temperature regimes simulated in CY and WY are characteristic for mid of April until end of August in 1990–2005 and are guided by near-surface temperatures measured at two sites of a large, slow-flowing river (Main), and in a nonstratified quarry pond (nature protection area Mainflingen, Germany) during 1990–2005 (HLUG 2010; Fig. 1). Temperature curves followed the equation (1) *y* = −0.0013*x*^2^+ 0.51*x*—*b*, with *b* = 22.7°C for CY and *b* = 25.2°C for WY, while *b* = 28.1°C in the WYF scenario. The prospective water surface temperature increase of maximal 2.9°C in July is based on the assumption that shallow lakes will heat up at the same or higher rate than during the last 40 years. For instance, shallow lakes in the Netherlands heated up by 0.042°C×yr^–1^ from 1961 to 2006 (Mooij et al. 2008) and the lakes Windermere, Esthwaite Water, and Loch Leven in United Kingdom by 0.04–0.05°C×yr^–1^ from 1976 to 2005 (Thackerey et al. 2010). Considering a comparative heating rate, temperature would have increased about 2.9°C in ∼2080. This scenario fits well with the modeled maximal temperature increase of 2–4°C in the epilimnion of the Ammersee (Germany) as predicted for 2100 (Danis et al. 2004).

Air temperature in the environmental cabinets was daily adjusted to the specific temperature gradient in 0.1°C steps and recorded (TL20 loggers, AMZ Großhandels KG, Mainhausen, Germany) at 20-minute time interval. Simulated day length coincided with the natural day length in Frankfurt am Main and was weekly adjusted in 15–20 min steps. The irradiation period increased from 13.5 h light d^–1^ (mid of April) to 16.2 h light d^–1^ (end of June) and decreased to 15.0 h light d^–1^ (mid of August). Light was provided by fluorescent tubes (TL-D tubes, 18W/865, Philips GmbH, Hamburg, Germany). Light intensity was set to 6400 Lux and relative air humidity to 60%.

Preparation and experimental implementation for a single generation was conducted as described for the life-cycle toxicity test with slight modifications. In short, merely 900-mL reconstituted water covered the sediment layer during water-sediment aging for 3–5 days. Furthermore, pyrimethanil was applied to L1-larvae in 100 mL portions (20 mg pyrimethanil L^–1^ reconstituted water). Besides, oviposition containers and wells with separated egg ropes were filled with either reconstituted water (control ropes) or 2 mg L^–1^ pyrimethanil solution. A new generation was started if any emergence could be observed for three days in the previous generation of the respective temperature regime. Ten well-developed egg ropes per treatment were pooled (only nine egg ropes for both F_4_ generations in WY, only four egg ropes for F_3_ control in WYF) and 60 larvae randomly transferred to each test vessel.

### Life-cycle parameters

Nonemerged midges were computed as percentage mortality per replicate. From emerged midges, sex ratio, development rate per day, and mean emergence time (EmT_50_) of males and females were calculated. For the calculation of development rate, experimental life-cycle duration as well as median age of larvae at day of insertion were considered. EmT_50_ of males and EmT_50_ of females were estimated to be the day when 50% of males or females emerged. Therefore, the natural logarithm (*x*) of time [day] was plotted against the number of normalized [%], cumulated, emerged male or female midges for each replicate, and nonlinear regression analysis was completed (logistic curve, maximal response = 100, GraphPad Prism®, version 5.01, Graph Pad software). In addition to the specifications of OECD guideline 219 (OECD 2004), four reproductive parameters of *C. riparius* were scrutinized. Median time until oviposition (time between median day of emergence and median time of breeding), number of eggs per egg mass (size of egg ropes), and sum of fertile or produced eggs (offspring and potential offspring) were determined for each replicate. Numbers of eggs per rope were counted before larvae hatched (for method see Vogt et al. 2007a) and fertility of eggs was scrutinized up to 8 days. A potential population growth rate (PGR) was computed for each replicate according to a simplified Euler–Lotka model and based to the calculation of Vogt et al. (2007b). The PGR calculated in this study accounts for the potential negative effects of small swarm size per replicate on fertility. Calculation of PGR as proposed by Vogt et al. (2007b) was therefore modified by the parameters potential offspring and median day until oviposition instead of offspring and EmT_50_ of females.

### Genetic diversity

In order to reveal potential effects of the chemical and temperature treatment on the level of genetic variability in the multigenerational study, expected (*H_E_*) and observed (*H*_0_) heterozygosity values of the source population and the final generations of all treatments were measured at five variable microsatellite loci (Nowak et al. 2006). For this purpose, 24 adults from the stock population and 18 individuals from each population of the final generation were taken and stored separately under dry conditions for genetic analysis. Laboratory procedure was performed as described in (Nowak et al. 2006, 2007a). In brief, DNA was extracted using a standard chloroform procedure. Microsatellite fragments were amplified via polymerase chain reaction and visualized by size separation on a 3730 DNA analyzer (Applied Biosystems, Foster City, CA). Alleles were scored with GeneMarker software (Softgenetics, State College, PA). *H_E_* and *H*_0_ values were calculated across loci, and chi-square test followed by Bonferroni correction was used to check for significant deviations from Hardy–Weinberg equilibrium (HWE). Population genetic parameters were calculated using Genalex software (Peakall and Smouse 2006).

### Pyrimethanil analysis

For pyrimethanil analysis, the test medium (1 mL) was sampled from all test vessels approximately 1 h after application and at the end of the experiment or generation, stored at −20°C, and purified (centrifugation at 13,000 rpm for 1 h and 0.2 μm filtration, Minisart RC 4, Satorius, Göttingen, Germany). In addition, ≥2.5 mg sediment was taken from the top layers (≥1 cm) at the end of each experimental generation and stored at −20°C. For pyrimethanil extraction, 2 g wet sediment was spiked with 4 mL ethyl acetate (>99.9% pure). This blend was bathed in ultrasonic sound for 15 min, strongly admixed and centrifuged at 4400 rpm for 10 min. Supernatant was spiked with 500-μl dimethyl sulfoxide (>99.8% pure) and evaporated under nitrogen flow. Sediment extracts were directly filtered (0.2 μm, Minisart SRP, Satorius, Germany) into high pressure liquid chromatography (HPLC) vials.

Qualification and quantization of pyrimethanil was conducted by HPLC using a C18 column (precolumn 4.3 × 10 mm, main column 4.3 × 150 mm, 5-μm particle size, 120-Å pore size, Acclaim 120, Dionex, Idstein, Germany) and an HPLC-UV system (software Chromeleon Version 6.60 SP2 build 1472, Dionex). The applied isocratic method operated with 40% methanol (A)/60% pure water (B) as a mobile phase (1 mL×min^–1^). After injection of 20 μl of sample, the mobile phase was gradually increased to 94% A/6% B within 18 min (25°C). Subsequently, the column was equilibrated at 40% A/60% B for 5 min. Retention time of pyrimethanil averaged to 14.5 min. Pyrimethanil concentrations were deduced from peak area relative to that of an internal standard at 254 nm. Calibration line (*n* = 3) was linearly correlated from 10 μg L^–1^ up to 50 mg L^–1^ pyrimethanil (Pestanal®, Sigma-Aldrich). Limit of detection was 34 ng L^–1^ (3.3-fold residual SD from linear regression). Limit of quantification was 3.3 μg L^–1^ (Eurachem approach: 0–21 μg L^–1^, *n* = 6, see Vial and Jardy 1999). Time-weighted mean of pyrimethanil concentration (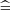 average actual concentration, AAC) in the test media was calculated as described by OECD guideline 211 (OECD 1998). Sediment recovery rate averaged to 68.3%.

### Statistical analysis

Life-cycle data are reported as mean ± standard deviation. In the life-cycle toxicity test, NOAEC and EC_50_/LC_50_ values (50% effective/lethal concentrations ± 95% confidence interval [CI]) were calculated from concentration–response relationships by means of nonlinear regression analysis (*x* = log(*x*), software GraphPad Prism®, version 5.01, GraphPad Software Inc., La Jolla, CA). To detect the NOAEC in the life-cycle toxicity test and to test for differences among ACC of pyrimethanil of three temperature scenarios, Tukey's multiple comparison test was performed, subsequent homogeneity of variances (Bartlett's test, *P* < 0.05) was proven and unifactorial Model I analysis of variance (ANOVA) (*F, P* < 0.05) accomplished (software GraphPad Prism®). Life-cycle toxicity test data were arcsine transformed in the case of percentage data and square root transformed if heterogeneous. Still nonparametric data were tested with Kruskal–Wallis test followed by Dunn's test (*P* < 0.05). Multigenerational life-cycle data were statistically tested either as raw or transformed data as specified in Table 2 (software Statistica, version 7.1, StatSoft, Inc., Hamburg, Germany). Homogeneity of variances was tested with Cochran's or Levine's test (*P* < 0.01, Table 2). Effects of independent variables pyrimethanil, temperature scenario, and time (generation) were estimated by a repeated measurement two-way ANOVA.

**Table 2:** Repeated two-way ANOVA of life-cycle parameter of *C. riparius* during a multigenerational study. Ten dependent (life-cycle parameter) and three independent variables (pyrimethanil treatment ‘ temperature scenario ’ generational time) were tested. Applied data transformations for statistical analysis are specified in brackets

Life-cycle parameter		P	T	Gen	P ′ T	P ′ Gen	T ′ Gen	P ′ T ″ Gen
	df	1	2	4	2	4	8	8
Mortality	F	0.50	1.92	45.95	1.48	2.94	11.99	1.10
[arcsine/square root]	p	0.49	0.17	<0.001	0.25	0.02	<0.001	0.37
				_^***^_		_*_	_^***^_	
Developmental rate	F	8.70	286.70	1271.50	10.10	7.70	890.70	3.90
[raw]	p	<0.01	<0.001	<0.001	<0.001	<0.001	<0.001	<0.001
		_^**^_	_^***^_	_^***^_	_^***^_	_^***^_	_^***^_	_^***^_
Proportion of emerged	F	0.62	25.77	6.53	0.83	0.63	9.36	1.56
Females	p	0.44	<0.001	<0.001	0.45	0.65	<0.001	0.15
[arcsine/square]			_^***^_	_^***^_			_^***^_	
Mean emergence time	F	0.59	1152.29	6.53	1.48	0.63	9.36	1.56
(EmT_50_) of females	p	0.45	<0.001	<0.001	0.25	0.65	<0.001	0.15
[raw]			_^***^_	_^***^_			_^***^_	
Mean emergence time	F	5.10	52039	47296	4.10	3.40	43071	1.50
(EmT_50_) of males	p	0.03	<0.001	<0.001	0.03	0.01	<0.001	0.18
[ln]			_^***^_	_^***^_	_*_	_*_	_^***^_	
Mean time until	F	1.93	26.16	15.27	1.10	4.94	6.04	0.74
Oviposition	p	0.18	<0.001	<0.001	0.35	<0.01	<0.001	0.65
[raw]			_^***^_	_^***^_		_^**^_	_^***^_	
Mean size of egg ropes	F	3.02	26.93	128.40	4.37	0.85	7.33	0.78
[square]	p	0.10	<0.001	<0.001	0.02	0.50	<0.001	0.62
			_^***^_	_^***^_	_*_		_^***^_	
Sum of fertile eggs	F	0.75	9.54	37.29	1.61	3.77	4.32	0.88
= offspring	p	0.40	<0.001	<0.001	0.22	0.007	<0.001	0.54
[square root]			_^***^_	_^***^_		_^**^_	_^***^_	
Offspring per female	F	1.50	16.72	15.27	2.27	4.94	6.04	0.74
[square root]	p	0.23	<0.001	<0.001	0.13	<0.01	<0.001	0.65
			_^***^_	_^***^_		_^**^_	_^***^_	
Potential growth rate	F	0.57	80.00	142.11	0.54	3.59	93.01	2.14
(PGR_pot_)	p	0.46	<0.001	<0.001	0.59	0.009	<0.001	0.04
[raw]			_^***^_	_^***^_		_^**^_	_^***^_	_*_

P = pyrimethanil; T = temperature scenario; Gen = generational time/thermal gradient; df = degree of freedom; F = model mean square: error mean square; p = probability; * = significant; ^**^ = highly significant; ^***^ = highest significant.

## Results

### Toxicity of pyrimethanil on C. riparius during one generation

The life-cycle toxicity test with *C. riparius* was valid according to the OECD guideline 219 (OECD 2004): oxygen saturation was ≥70%; pH ranged between 7.1 and 8.2; survival of controls was ≥72% and emergence took place between day 13 and 24 with 0.06 midges per day. The water chemistry did not show concentration-dependent developments (0–0.14 mg L^–1^ phosphate; 30–80 μg L^–1^ nitrite; 0–8 mg L^–1^ ammonium). EmT_50_ of untreated females averaged to 18.4 ± 0.99 days in controls and 18.2 ± 0.85 days in solvent controls. Proportion of emerged females (compared to males) amounted to 53 ± 14% in controls and 47 ± 7% in solvent controls. Females produced 8981 ± 3447 and 7551 ± 1824 eggs, 45 ± 16% and 56 ± 27% thereof were fertile. The calculated PGR was 1.21 ± 0.05 and 1.22 ± 0.05 (control/solvent control), respectively.

**Figure 2:**
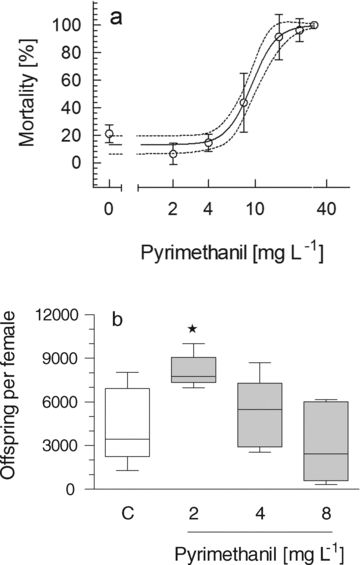
*Chironomus riparius* in the life-cycle toxicity test with pyrimethanil. (A) Mortality [%, mean ± SD]. (B) Offspring per female [number of fertile eggs per female ± 5/95 percentiles]. NOAEC = no-observed-adverse-effect concentration; LC_10_ = concentration causing 10% lethal effect; LC_50_ = concentration causing 50% lethal effect; C = control; asterisk = significant difference to control (*P* < 0.05).

For the endpoint mortality, a NOAEC of 4 mg L^–1^ and an LC_50_ of 9.27 [CI 8.20–10.47] mg L^–1^ pyrimethanil were calculated on the basis of a clear concentration–response relationship using nominal concentrations (NOM, Fig. 2A). The respective lethal concentrations based on AAC for mortality were: NOAEC = 2.83 mg L^–1^ and LC_50_ = 5.95 [CI 5.41–6.55] mg L^–1^ pyrimethanil. At the lower range of tested pyrimethanil concentrations, development and reproduction were less affected than survival. The estimation of effective concentrations for the endpoint emergence time was not possible as regressions were too steep. EmT_50_ was slightly reduced until 24 mg L^–1^ pyrimethanil (16.2 ± 3.11 days at 24 mg L^–1^ [NOM] ≙ 19.6 mg L^–1^ [AAC]; 15.0 days at 32 mg L^–1^ [NOM] ≙ 21.7 mg L^–1^ [AAC]) albeit EmT_50_ did not differ in variance at all. The development rate of 0.06 midges per day was unaffected up to 8 mg L^–1^ pyrimethanil (5.32 mg L^–1^ [AAC]).

Analysis of reproductive endpoints led to a NOAEC of 8 mg L^–1^ [NOM] (≙ 5.32 mg L^–1^ [AAC]), while reproduction was nearly entirely inhibited at 16 mg L^–1^ [NOM] (≙ 9.07 mg L^–1^ [AAC]). It has to be noted, however, that both the total and fertile egg production per female were significantly enhanced by 2 mg L^–1^ pyrimethanil [NOM] (≙ 1.36 mg L^–1^ AAC) compared to controls (*P* < 0.05, Fig. 2B). The PGR evidenced a concentration–response relationship with an AAC-corrected NOAEC of 5.32 mg L^–1^ and EC_50_ of 13.1 [CI 8.09 to 21.3] mg L^–1^ pyrimethanil.

### Interactive effects of pyrimethanil and temperature during consecutive generations

#### Water chemistry

In the test media, pH averaged to 7.6 ± 0.4 and oxygen saturation to 88.8 ± 11.1% over all generations and scenarios, respectively. After completion of a generation, phosphate varied between 0 and 0.3 (CY), 1.84 (WY), or 3 (WYF) mg L^–1^, while conductivity ranged from 595 to 633 μS cm^–1^ (CY), 494 to 729 μS cm^–1^ (WY), or 513 to 918 μS cm^–1^ (WYF). Nitrite amounted on average to 0.11 ± 0.19 mg L^–1^, nitrate to 5.0 ± 6.1 mg L^–1^, and ammonium to 4.5 ± 3.2 mg L^–1^. Pyrimethanil in the test media (AAC) averaged over all generations to 1.20 ± 0.07 mg L^–1^ (CY), 1.38 ± 0.15 mg L^–1^ (WY), or 1.11 ± 0.23 mg L^–1^ (WYF) and pyrimethanil in the upper sediment layers at the end of generations to 0.23 ± 0.14 μg g^–1^ (CY), 0.33 ± 0.25 μg g^–1^ (WY), and 0.22 ± 0.09 μg g^–1^ (WYF). The mean AAC in the WY scenario was significantly higher than in the CY and WYF scenario (*P*≤ 0.001), while the AAC per generation (Table 1) revealed no correlation with the mean temperature per generation (*R*^2^≤ 0.19). Pyrimethanil content in the upper sediment layers did not differ within and among three scenarios (*P* > 0.05; Table 1).

**Table 1:** Mean temperature (°C), median time-weighted pyrimethanil concentration in the water phase [mg L^–1^], and median pyrimethanil concentration in the sediment (μg g^–1^) ± SD for each generation (F_0_–F_4_) in three temperature scenarios CY, WY, and WYF simulated in the multigenerational study

		CY	WY	WYF
Mean temperature	F_0_	15.21 (± 2.12)	17.34 (± 1.7)	20.14 (± 1.65)
	F_1_	19.96 (± 0.77)	21.82 (± 0.86)	24.62 (± 0.99)
	F_2_	21.7 (± 0.3)	23.76 (± 0.39)	26.92 (± 0.36)
	F_3_	22.02 (± 0.11)	24.56 (± 0.16)	27.97 (± 0.22)
	F_4_	20.65 (± 0.7)	24.27 (± 0.58)	x
Pyrimethanil in surface water (TWA)	F_0_	1.02 ± 0.08	0.96 ± 0.07	1.56
	F_1_	1.22 ± 0.32	0.96	1.35
	F_2_	0.92 ± 0.27	1.38 ± 0.08	1.03
	F_3_	1.76 ± 0.36	1.16	1.55 ± 0.30
	F_4_	1.83	0.73	
	Mean	1.20 ± 0.07^a^	1.38 ± 0.15^b^	1.11 ± 0.23^a^
Pyrimethanil in the upper sediment layer	F_0_	0.19 ± 0.04	0.91	0.21 ± 0.06
	F_1_	x	0.31 ± 0.11	x
	F_2_	0.15 ± 0.03	0.45 ± 0.22	0.26 ± 0.10
	F_3_	0.41 ± 0.14	x	0.19 ± 0.07
	F_4_	0.12 ± 0.07	0.12 ± 0.08	
	Mean	0.23 ± 0.14	0.33 ± 0.25	0.22 ± 0.09

CY = simulation of a cold growing season in 1990–2005; WY = simulation of a warm season in 1990–2005; WYF = simulation of a projected warm season in 2050–2080. *n* (pyrimethanil generation^–1^) = 1 or 6; x = samples lost; uppercase letters = significant differences (Tukey post test).

#### Life history

Within the experimental period mid of April until end of August, *C. riparius* generated five generations in the simulations CY and WY within 139 and 125 days, respectively (Fig. 1). Four generations were produced under the WYF scenario within 90 days but insufficient hatching of agile larvae impeded the establishment of a fifth generation with full replicability. Life-cycle parameters were mainly controlled by temperature scenario and temperature gradient, the latter being inseparable from number of generations and dynamic light duration (Table 2). The two main factors caused highly significant, individual, and interactive effects on almost all life-cycle parameters of *C. riparius*. The concentration of 2 mg pyrimethanil L^–1^ had a weak impact on life-cycle parameters that significantly altered over time. The interaction pyrimethanil ‘ generation affected seven of 10 studied life-cycle parameters, whereas pyrimethanil ’ temperature scenario caused interactive effects on only three of 10 life-cycle parameters related to emergence and reproduction. Merely the endpoints development rate and PGR were interactively influenced by pyrimethanil ‘ temperature ’ generation.

**Figure 3:**
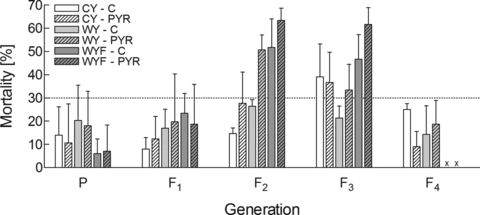
Mortality [%, mean ± SD] of *C. riparius* after exposure to control conditions (uniform bars) or 2 mg L^–1^ pyrimethanil (striped bars) during consecutive generations at three dynamic temperature regimes. CY = simulation of a cold growing season in 1990–2005 (white bars); WY = simulation of a warm season in 1990–2005 (light gray); WYF = simulation of a projected warm season in 2050–2080 (dark gray); C = control treatment; PYR = pyrimethanil treatment; F_0_–F_5_ = consecutive generations; x = no data.

**Figure 4:**
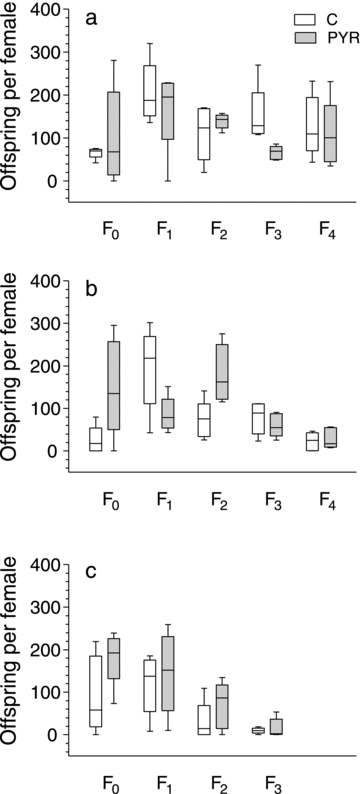
Offspring per female [number of fertile eggs per female ± 5/95 percentiles] of *C. riparius*. Offspring per female is produced by either control (white boxes) or 2 mg L^–1^ pyrimethanil (gray boxes) treated midges during consecutive generations (F_0_–F_5_) under simulation of (a) a typical cold year in 1990–2005 (CY), (b) a warm year in 1990–2005 (WY), or (c) a temperature regime expected for a warm year in 2050–2080 (WYF).

**Figure 5:**
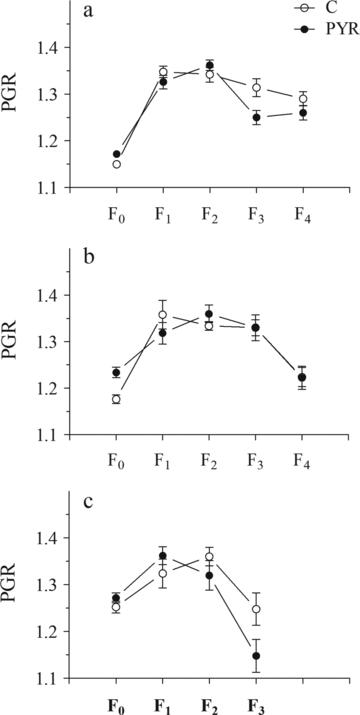
Potential population growth rate [PGR ± 5/95 percentiles] of *C. riparius*. PGR over consecutive generations (F_0_–F_5_) is depicted either if exposed to control conditions (white boxes) or 2 mg L^–1^ pyrimethanil (gray boxes) under simulation of (A) a typical cold year in 1990–2005 (CY), (B) a warm year in 1990–2005 (WY), or (C) a temperature regime expected for a warm year in 2050–2080 (WYF).

Temperature and pyrimethanil caused most conclusive effects on mortality, the number of fertile eggs, and the PGR (Figs. 3–5); and are therefore described in the following, while other endpoints are presented in the Appendix. Lethal effects on *C. riparius* were most pronounced in the future scenario as maximum control mortality was 39% in CY (F_3_ generation) and 26% in WY (F_3_ generation), but 52% in WYF (F_2_ generation; Fig. 3). Furthermore, mortality of *C. riparius* in F_4_ decreased only in CY and WY after reaching its maximum in F_3_. Under pyrimethanil exposure in the CY scenario, a 13% higher mortality than in controls was observed in the F_2_ generation. This difference between treatments vanished in the following F_3_ generation and reversed in the F_4_ generation, where control exhibited a 16% higher mortality than the fungicide treatment. Pyrimethanil-induced mortality under scenario WY was twice as high (50%) as control mortality (26%) during F_2_ generation, while this negative effect withered as well in the following generations (14% higher PYR mortality in F_3_, 4% higher PYR mortality in F_4_). In the warmest scenario WYF, mortality increased up to 62–63% in pyrimethanil treatments during F_2_ and F_3_ generation, although high temperature caused a likewise high control mortality of 47–52%.

The size reduction of egg ropes with increasing generational time and temperature was mirrored by a reduced sum of fertile eggs in both warm year scenarios, but not in the CY scenario (Fig. 4; Table A1). An additional pyrimethanil exposure insignificantly increased the sum of fertile eggs per female in 50% of overall generations (CY: F_0_ and F_1_ generation, WY: F_0_ and F_2_ generation, WYF: F_0_, F_1_, and F_2_ generation; Fig. 4), while pyrimethanil slightly reduced the amount of fertile eggs per female in the CY F_3_ generation and F_1_ and F_3_ generation in the WY simulation (Fig. 4A–C).

The PGR followed the temperature dynamics and was most strongly influenced by temperature scenarios and generational time (Fig. 5A–C; Table 2). Minimal PGR of 1.15 was discerned in controls of CY F_0_ generation and in the pyrimethanil treatment of the final F_3_ generation within WYF scenario. Maximal PGR in control populations counted to 1.33–1.36 in three scenarios (CY F_2_ generation, WY F_1_, F_2_, and F_3_ generations, WYF F_2_ generation). Effects of pyrimethanil exposure on PGR were ambiguous. On the one hand, pyrimethanil slightly reduced the PGR in the CY F_3_ generation by 6.7% and in the WYF F_3_ generation by 8.0%. On the other hand, pyrimethanil slightly enhanced the PGR in the WY F_0_ generation by 4.9%.

### Genetic diversity

In the source population, all loci showed considerable allelic variation with *H_E_* and *H*_0_ values of 0.59 and 0.58, respectively (Table 3). Similar values were obtained under control conditions after four-to-five generations (*H_E_* = 0.55–0.65). Measured heterozygosity values in the pyrimethanil-exposed populations, in contrast, were lower than the respective values in the controls. Lowest *H_E_* and *H*_0_ values were obtained for the pyrimethanil-treated population under the warm year future scenario (*H*_0_ = 0.35; *H_E_* = 0.46), which translates into a 41% (*H*_0_) and 21% (*H_E_*) loss of allelic variation compared to the source population. After four-to-five generations, a significant deviation from HWE was found in two control treatments (CY, WY) at a single locus, and at three loci in the pyrimethanil-treated WYF population.

**Table 3:** Genetic diversity of *C. riparius* at five microsatellite loci during the multigenerational study. Observed (*H*_0_) and expected heterozygosity (*H_E_* = gene diversity) values are shown

	Source	CY-C	CY-PYR	WY-C	WY-PYR	WYF-C	WYF-PYR
**H_0_**	**0.59**	**0.55^1^**	**0.43**	**0.65^1^**	**0.54**	**0.60**	**0.35**^3^
% of source pop.	92.6	73.2	109.6	90.8	102.6	59.5	
% loss to source pop.	7.4	26.7	−9.6	9.2	−2.6	40.5	
% loss to control		21.5		17.6		41.6	
**H_E_**	**0.58**	**0.50**	**0.56**	**0.58**	**0.54**	**0.53**	**0.46**
% of source pop.	85.3	95.7	98.5	91.7	89.4	78.7	
% loss to source pop.	14.7	4.3	1.5	8.3	10.6	21.3	
% loss to control		−12.7		6.9		12.5	

1 Superscript letters indicate the number of loci with significant deviation from Hardy–Weinberg equilibrium after Bonferroni correction.

## Discussion

The present study highlighted the importance of climate change research, including multiple endpoints in toxicity testing to better understand the ecotoxicological risk of low doses of agrochemicals arising in the future. At first, the results of the multigeneration study provided clear evidence that GCC conditions pose a high risk for *C. riparius* populations. Second, adverse effects on *C. riparius* observed under future climate simulation became strengthened by a supplement influence of a regulatory approved NOAEC/2 of the fungicide pyrimethanil. Although the fungicidal impact on life-history parameters was relatively weak over multiple generations, the strongly degraded genetic diversity under contemporary thermal and fungicidal stress might reduce the resilience of *C. riparius* against forthcoming stressful environments.

Temperature has been ascribed a major role in the ecology of chironomids (Larocque–Tobler et al. 2010). Other factors such as the degree of genetic variation (Vogt et al. 2007c; Oetken et al. 2009), larval density (Hooper et al. 2003), food supply (Péry and Garric 2006), photoperiod (Danks 1978; Ineichen et al. 1979), and oxygen level (McFarlane and McLusky 1972; Airas et al. 2008) strongly influence the thermal and/or xenobiotic response of chironomid populations. For instance, developmental time of *C. riparius* was similar at limiting and ad libitum food conditions at 15°C, but not at 20°C and temperatures above (Péry and Garric 2006). In addition, genetically impoverished *C. riparius* populations were more susceptible to tributyl tin (TBT) at increasing temperature rather than genetically diverse populations (Oetken et al. 2009). To avert those limitations from our multigeneration study, we initially exposed a genetically rich source population of *C. riparius* at low larval density and provided ad libitum larval food with precautionary food buffer in the sediment, naturally adapted photoperiods, and a high oxygen saturation over the experimental period (cf. Hooper et al. 2003; Charles et al. 2004; Nowak et al. 2007b; Vogt et al. 2007c; Airas et al. 2008). At this setting favorable for population growth, life-history traits of the ectotherm midge followed, as expected, the shifting temperature gradients. The higher the temperature was, the higher the mortality, the faster the emergence, and the stronger the depression of reproduction and population growth (Figs. 3–5; Table A1). These findings generally support several previous studies with *C. riparius*, with few deviations (Péry and Garric 2006; Vogt et al. 2007c; Oetken et al. 2009).

Emergence time of *C. riparius* is slightly reduced at near-natural temperature conditions if compared to EmT_50_ values reported for genetically rich populations after constant temperature exposure (Table A1; Vogt et al. 2007c; Oetken et al. 2009). The accelerated development under fluctuating temperature is well known as Kaufmann's effect or thermal hormesis (Kaufmann 1932; Keen and Parker 1979). The high mortality and failure of *C. riparius* to effectively reproduce at a mean temperature of 26.9–28.0°C during F_2_/F_3_ WYF generations were surprising (Table 1; Fig. 3). At 26.7°C, no effect on survival and only a slight decrease in egg production was observed in the study of Péry and Garric (2006). However, while we exposed eggs and L1-larvae, Péry and Garric (2006) exposed only L4-larvae. An age-dependent thermotolerance (Frouz et al. 2002; Bowler and Terblanche 2008) and the generally high susceptibility of early larval stages in *Chironomus* may explain these observed differences. Likewise surprising is the high control mortality (39%) in the F_3_ CY generation, while the mean air temperature of 22.02 and a day–night temperature variation of 0.13–0.22°C (SD) should not be stressful for *C. riparius* (OECD 2004). Since the water chemistry was in a good shape during the F_3_ CY generation, the high control mortality is probably based on the loss of genetic diversity (Vogt et al. 2007b), although the F_4_ CY generation should not have been recovered in that case. However, a similar high control mortality at the fourth *C. riparius* generation and a subsequent recovery was observed at 20 ± 1°C by Vogt et al. (2007a) and might be an inherent response pattern of *C. riparius*. The discrepancies in thermal responses underpin that thermal effects on early life-history stages should be included to describe the thermal response of chironomid populations comprehensively. Moreover, thermal hormesis and individual responses of distinct generations should be kept in mind if interpreting constant-temperature life-cycle tests (Keen and Parker 1979).

The monthly July mean near-surface temperature (*T*_Jul_) strongly correlates with the abundance and species composition of chironomids (Larocque–Tobler et al. 2010). Hence both present-day scenarios, in particular the warm year present-day scenario, provide favorite thermal environments for temperate *C. riparius* populations (Figs. 1 and 3–5). During the last three to five decades, however, *T*_Jul_ of more than a few northern hemisphere lakes has increased by approximately 0.001–0.009°C yr^–1^ and in selected shallow lakes of the Netherlands and in the United Kingdom by even 0.04–0.05°C yr^–1^ (Scheffer et al. 2001; Mooij et al. 2008; Adrian et al. 2009; Thackeray et al. 2010). Regional models for Germany predict a mean air temperature increase in summer by 2.7–4.1°C in 2071–2100 (Jacob et al. 2008). Thus, temperate, particularly shallow water bodies may heat up by approximately 1.4–2.9°C in 2100 if calculating a 50–70% increase of air temperature (Kristensen 2008) or by ∼3.7°C if considering an ascending slope of 0.042°C *T*_Jul_ yr^–1^. Under such a thermally polluted situation (WYF scenario), population dynamics of *C. riparius* were severely deteriorated. After exposure to a critical temperature above the thermoneutral zone as marked by an elevated mortality during two consecutive WYF generations, insufficient number of agile larvae were released to establish a fifth experimental generation with full replicability (Figs. 3, 4C, and 5C). Nonetheless, a small number of larvae with an unaffected genetic diversity were released from the F_3_ WYF control population and could insure the population from extinction (PGR > 1; Fig. 5C; Table 3).

The pyrimethanil concentration causing a beneficial effect on *C. riparius* reproduction in the life-cycle toxicity test provoked slight-to-moderate and ambivalent life-history effects at near-natural temperature conditions as simulated in the multigeneration study (Figs. 1 and 3–5; Table A1). A trend for stimulating effects of pyrimethanil was detected in the multigenerational study, mostly in view of reproductive parameters (Fig. 4; Table A1). A stimulation of reproduction in response to chemicals was likewise observed in two earlier multigeneration studies and may reflect a general stress reaction of *C. riparius* to low doses of pollutants (Ristola et al. 2001; Vogt et al. 2007a). At first glance, stimulating effects on reproduction, particularly in the beginning of the reproductive season, would be beneficial for population growth in the long term. But hormesis is not mandatorily positive, given that an overcompensation of xenobiotically interfered homeostasis by way of increased activity of defense and repair mechanisms (e.g., antioxidant/DNA repair capacity) is energetically expensive (Costantini et al. 2010). To maintain the homeostasis, defense mechanisms are activated at very low xenobiotic concentrations long before life-cycle parameters response, which is especially true for *C. riparius* (Choi et al. 2002; Park et al. 2009). Hence, an observed stimulatory effect of pyrimethanil on the life cycle of *C. riparius* at a comforting situation such as in a life-cycle toxicity test or at simulated spring conditions should diminish or convert into a harmful effect over several generations due to a cumulative energetic imbalance, in particular under stressful temperature conditions.

In fact, harmful effects of the low doses of pyrimethanil were rarely detectable with the monitoring of life-cycle reactions of *C. riparius* as they often depend on generation number (Figs. 3–5; Tables 2 and A1). Inhibiting and stimulating pyrimethanil effects on survival, emergence, and reproduction often cancel each other, resulting in a neutral population growth (Figs. 3–5; Table A1). Furthermore, adverse pyrimethanil effects on survival of *C. riparius* in two present-day scenarios vanish until the end of the summer, which probably hints to an adaptive response (Fig. 3). Especially peculiar is the stimulation of survival in the CY F_4_ generation that contradicts the harmful effect obvious in the F_2_ generation. Adaptation of metabolic systems to CY and WY summer conditions may have reduced the overall stress level, while in the CY F_4_ generation an overcompensation toward mild chemical stress may have occurred (Heugens et al. 2001; Calabrese 2010). However, surprisingly strong effects of the fungicide were disclosed at a rather cryptic parameter, namely genetic variability. Pyrimethanil-exposed populations showed generally lower heterozygosity values compared to the respective control treatments (Table 3). These results corroborate the belief that a pollution-induced loss of genetic diversity can take place within few generations (Vogt et al. 2010).

The considerable risk of the NOAEC/2 of the fungicide for the biological model became apparent under GCC conditions respecting the increased mortality, reduced population growth, and the striking loss of genetic diversity of *C. riparius* (Figs. 3 and 5; Table 3). Although few larvae were still released from the WYF F_3_ generation, a drop of >40% of the initial heterozygosity was measured within only four generations. The rapid decrease of allelic variation in the WYF pyrimethanil treatment is likely a side effect of the high mortality and the consequently reduced swarm size. This reduction of individuals contributing to the next generations led to increased inbreeding rates and increased genetic drift effects, resulting in lowered rates of heterozygosity. The extremely lowered effective population size of *C. riparius* attributable to mild fungicidal stress in the future scenario also explained the increased number of loci departing from HWE, caused by strong genetic drift effects in small populations. The effects of genetic erosion for the long-term viability of a population are not exactly predictable, but there is a general agreement that a reduced genetic diversity will lead to a lowered adaptation potential to changing environmental conditions and is frequently accompanied by reduced fitness due to inbreeding depression (Nowak et al. 2007b; Brown et al. 2009; Fox and Reed 2011). At the same time, high genetic variation is needed to allow species to cope with environmental perturbations more flexibly and thereby insure populations against inevitable environmental changes. Thus, interaction of chemical and thermal stress may severely reduce the fitness of *C. riparius* populations in the future, particularly once they are affected by novel stressors.

The results highlighted the importance of climate change research including multigenerational effects and multiple endpoints to better understand and manage the ecotoxicological risk of low-dosed agrochemicals under GCC. The present study may help to understand more broadly how aquatic species cope with low doses of agrochemicals at near-natural temperature regimes in general and at GCC conditions in particular, and may provide a framework for further studies. Multigeneration studies and the simulation of natural (climatic) conditions allow for more realistic assessments and reveal, in combination with extensive life-history assessments and measures of genetic diversity, otherwise concealed effects. Our results indicate that not only the impact of climate change, but also low concentrations of pesticides might pose a reasonable risk for aquatic insects in future. Therefore the ecotoxicological community should adduce evidence that uncertainty factors used by ERA are still protective for aquatic environments under climate change conditions to provide a maximum level of protection for aquatic biodiversity and ecosystem health.

## Appendix

The appendix presents the developmental and additional reproductive endpoints as examined during the multigeneration study as well as the calculation of the population growth rate after Vogt et al. (2007a).

Increasing temperature accelerated the development of *C. riparius* (Table A1). Life-cycle duration was shorter or equal in pyrimethanil treatments, only the pyrimethanil treatment in F_4_-generation of CY scenario had a 1.5 days longer life cycle than the control (Table A1). Developmental rate in pyrimethanil treatments was considerably depressed in F_3_ and F_4_ generation in WY (0.006 and 0.005 less emerged midges per day) if compared to controls. Sex ratio and EmT_50_ of females were not influenced by pyrimethanil, although there was a trend toward prolonged emergence time of females in F_3_ and F_4_ generation of CY, in F_1_ and F_4_ generation of WY, and F_1_ generation of WYF scenario (Tables 2 and A1).

**Table A1:** Life-cycle parameter of controls or pyrimethanil-treated *C. riparius* at three dynamic temperature regimes CY, WY, and WYF examined during consecutive generations (F_0_–F_5_). CY = simulation of a cold growing season in 1990–2005; WY = simulation of a warm season in 1990–2005; WYF = simulation of a projected warm season in 2050–2080; C = control; P = pyrimethanil

		CY				WY				WYF			
				
		C		PYR		C		PYR		C		PYR	
Development rate	F_0_	0.030	± 0.001	0.030	± 0.002	0.036	± 0.001	0.036	± 0.001	0.042	± 0.002	0.043	± 0.001
	F_1_	0.043	± 0.002	0.043	± 0.002	0.047	± 0.001	0.046	± 0.002	0.053	± 0.001	0.052	± 0.001
	F_2_	0.054	± 0.002	0.055	± 0.001	0.053	± 0.002	0.055	± 0.001	0.054	± 0.001	0.054	± 0.000
	F_3_	0.052	± 0.002	0.050	± 0.002	0.059	± 0.002	0.053	± 0.002	0.061	± 0.002	0.061	± 0.001
	F_4_	0.049	± 0.002	0.050	± 0.002	0.054	± 0.001	0.049	± 0.002	x		x	
Female fraction	F_0_	0.51	± 0.07	0.49	± 0.03	0.51	± 0.06	0.59	± 0.07	0.49	± 0.05	0.44	± 0.06
	F_1_	0.57	± 0.03	0.53	± 0.05	0.49	± 0.09	0.53	± 0.08	0.48	± 0.08	0.53	± 0.01
	F_2_	0.46	± 0.06	0.39	± 0.13	0.52	± 0.10	0.48	± 0.09	0.51	± 0.11	0.49	± 0.05
	F_3_	0.55	± 0.07	0.53	± 0.09	0.57	± 0.08	0.48	± 0.07	0.40	± 0.07	0.46	± 0.08
	F_4_	0.54	± 0.06	0.53	± 0.04	0.50	± 0.06	0.52	± 0.08	x		x	
EmT_50_ of females	F_0_	34.18	± 1.67	33.55	± 1.67	28.14	± 1.02	27.94	± 0.93	24.57	± 1.05	23.87	± 0.42
	F_1_	23.63	± 0.90	24.03	± 0.90	21.55	± 0.67	22.47	± 0.82	19.17	± 0.56	19.53	± 0.72
	F_2_	19.26	± 0.66	18.81	± 0.28	19.39	± 0.55	18.70	± 0.37	18.90	± 0.49	18.93	± 0.30
	F_3_	19.90	± 0.76	21.01	± 0.64	17.97	± 0.52	17.56	± 1.08	17.63	± 0.95	16.82	± 0.93
	F_4_	21.55	± 1.23	22.68	± 1.09	19.17	± 0.24	20.70	± 1.02	x		x	
EmT_50_ of males	F_0_	32.25	± 1.76	31.86	± 2.20	27.39	± 1.25	27.19	± 0.89	23.46	± 1.17	22.75	± 0.48
	F_1_	22.70	± 0.87	23.39	± 1.14	20.91	± 0.55	21.68	± 0.94	18.41	± 0.12	18.43	± 0.04
	F_2_	17.80	± 0.63	17.72	± 0.44	18.11	± 0.85	17.80	± 0.26	18.32	± 0.12	18.26	± 0.15
	F_3_	18.01	± 0.51	19.71	± 1.11	15.88	± 0.43	15.95	± 1.03	15.74	± 0.46	15.46	± 0.12
	F_4_	18.85	± 0.73	19.85	± 0.53	17.14	± 0.37	18.75	± 1.58	x		x	
Mean time until oviposition	F_0_	5.62	± 1.95	4.45	± 1.26	4.86	± 0.73	3.56	± 0.80	3.83	± 1.31	3.53	± 1.12
	F_1_	2.97	± 0.61	3.07	± 1.27	2.65	± 0.77	2.93	± 1.48	2.33	± 0.78	2.47	± 0.88
	F_2_	2.94	± 0.78	2.29	± 0.58	3.51	± 0.54	2.60	± 0.17	2.80	± 0.56	1.77	± 0.84
	F_3_	2.60	± 1.21	2.09	± 1.02	2.73	± 1.08	3.84	± 0.97	1.27	± 1.52	2.38	± 0.63
	F_4_	2.75	± 0.47	2.32	± 1.15	2.98	± 0.97	3.33	± 1.16	x		x	
Mean size of egg ropes	F_0_	744.3	± 109.3	794.3	± 67.2	618.0	± 153.2	711.1	± 78.3	682.2	± 91.3	609.7	± 39.3
	F_1_	669.3	± 37.0	695.1	± 50.0	666.0	± 23.7	662.6	± 56.2	594.2	± 122.8	589.2	± 41.3
	F_2_	533.6	± 23.8	608.4	± 36.2	566.4	± 64.3	675.9	± 52.9	673.1	± 56.9	663.2	± 43.6
	F_3_	489.2	± 17.5	501.1	± 68.9	379.5	± 115.1	392.6	± 86.2	244.6	± 148.5	174.9	± 163.9
	F_4_	463.1	± 33.1	473.9	± 98.2	255.0	± 107.3	282.2	± 115.9	x		x	
Sum of fertile eggs	F_0_	3518	± 1385	4833	± 4242	1469	± 2272	6354	± 4451	4976	± 4515	9438	± 3554
	F_1_	10258	± 3363	9237	± 5332	9706	± 5057	4633	± 3243	5290	± 3296	7414	± 5681
	F_2_	5661	± 2991	6228	± 1511	2947	± 1882	5205	± 1669	902	± 1295	1672	± 1410
	F_3_	5468	± 2418	2499	± 596	3747	± 1249	2676	± 978	610	± 510	528	± 463
	F_4_	443	± 33	452	± 99.1	237	± 107	263	± 115	X		x	
Standard growth rate^1^	F_0_	1.12	± 0.01	0.91	± 0.51	0.67	± 0.61	0.96	± 0.54	0.96	± 0.54	1.24	± 0.02
	F_1_	1.25	± 0.02	0.99	± 0.56	1.26	± 0.05	1.20	± 0.04	1.24	± 0.08	1.25	± 0.08
	F_2_	1.25	± 0.05	1.28	± 0.02	1.22	± 0.04	1.27	± 0.02	0.70	± 0.64	0.95	± 0.53
	F_3_	1.25	± 0.03	1.19	± 0.01	1.25	± 0.05	1.23	± 0.04	0.89	± 0.50	0.86	± 0.49
	F_4_	1.23	± 0.04	1.21	± 0.04	0.92	± 0.52	1.15	± 0.05	X		x	

1 Standard population growth rate (PGR) based on mortality, offspring per emerged female, proportion of emerged females, and EmT_50_ of females was computed for each replicate according to a simplified Euler–Lotka model as described in Vogt et al. (2007a).

Reproductive endpoints were likewise mainly influenced by temperature scenario and generation number, while pyrimethanil effects were weak (Table 2). Though the oviposition of pyrimethanil-treated *C. riparius* outspeeded that of controls in the F_0_ and F_2_ generation under CY and WY scenario (CY: −2.1 and −1.0 days; WY: −1.3 and −0.9 days) and the F_2_ generation under WYF scenario (-1.0 day) (Table A1). Then again, median time until oviposition was prolonged in pyrimethanil-treated animals of the F_3_ generation under WY and WYF scenarios (+1.1 day). Female midges produced on average more egg ropes in the simulated CY (Table A1). Per female, 0.8 ± 0.3/0.8 ± 0.6 egg ropes (control/pyrimethanil) were deposited in CY, 0.6 ± 0.2/0.7 ± 0.2 egg ropes (control/pyrimethanil) in WY, and 0.6 ± 0.2/0.7 ± 0.3 egg ropes (control/pyrimethanil) in WYF. Production of egg ropes by pyrimethanil-treated midges was advanced in F_3_ generation of the CY scenario and in F_0_ generation of the WY and WYF scenarios.

Mean size of egg ropes was largest in the CY scenario with 744 eggs (control) or 794 eggs (pyrimethanil) per rope in the F_0_ generation (Table A1). Egg ropes produced in the F_0_ generation of WY contained on average only 618 eggs (control) or 711 eggs (pyrimethanil), while egg ropes of WYF comprised only 682 eggs (control) or 610 eggs (pyrimethanil). Over the experimental period, mean size of egg ropes generally decreased to 38%/40% (control/pyrimethanil) in CY, 59%/60% (control/pyrimethanil) in WY, and 61%/71% (control/pyrimethanil) in WYF scenario.
